# A New Method for Tungsten Oxide Nanopowder Deposition on Carbon-Fiber-Reinforced Polymer Composites for X-ray Attenuation

**DOI:** 10.3390/nano13233071

**Published:** 2023-12-03

**Authors:** Marian Mogildea, George Mogildea, Sorin I. Zgura, Doina Craciun, Natalia Mihăilescu, Petronela Prepelita, Laura Mihai, Marian C. Bazavan, Vasile Bercu, Leonard Constantin Gebac, Raluca Maier, Bogdan S. Vasile, Valentin Craciun

**Affiliations:** 1 Institute of Space Science, 077125 Magurele, Romania; marian_mogildea@spacescience.ro (M.M.); szgura@spacescience.ro (S.I.Z.); 2 National Institute for Laser, Plasma and Radiation Physics, 077125 Magurele, Romania; doina.craciun@inflpr.ro (D.C.); natalia.serban@inflpr.ro (N.M.); petronela.garoi01@gmail.com (P.P.); laura.mihai@inflpr.ro (L.M.); 3 Faculty of Physics, University of Bucharest, 077125 Magurele, Romania; m_bazavan@yahoo.com (M.C.B.); vasile.bercu@unibuc.ro (V.B.); leonard.gebac@unibuc.ro (L.C.G.); 4 Romanian Research & Development Institute for Gas Turbines, 061126 Bucharest, Romania; raluca.maier@comoti.ro; 5 National Research Center for Micro and Nanomaterials, Bucharest National Polytechnic University of Science and Technology, 060042 Bucharest, Romania; vasilebogdanstefan@yahoo.com; 6 Research Center for Advanced Materials, Products and Processes, University of Bucharest, 060042 Bucharest, Romania; 7 Extreme Light Infrastructure for Nuclear Physics, Horia Hulubei National Institute of Physics and Nuclear Engineering, 077125 Magurele, Romania

**Keywords:** plasma, microwaves, X-ray attenuation, nanopowders, WO_3_

## Abstract

A new method for the synthesis and deposition of tungsten oxide nanopowders directly on the surface of a carbon-fiber-reinforced polymer composite (CFRP) is presented. The CFRP was chosen because this material has very good thermal and mechanical properties and chemical resistance. Also, CFRPs have low melting points and are transparent under ionized radiation. The synthesis is based on the direct interaction between high-power-density microwaves and metallic wires to generate a high-temperature plasma in an oxygen-containing atmosphere, which afterward condenses as metallic oxide nanoparticles on the CFRP. During microwave discharge, the value of the electronic temperature of the plasma, estimated from Boltzmann plots, reached up to 4 eV, and tungsten oxide crystals with a size between 5 nm and 100 nm were obtained. Transmission electron microscopy (TEM) analysis of the tungsten oxide nanoparticles showed they were single crystals without any extended defects. Scanning electron microscopy (SEM) analysis showed that the surface of the CFRP sample does not degrade during microwave plasma deposition. The X-ray attenuation of CFRP samples covered with tungsten oxide nanopowder layers of 2 µm and 21 µm thickness was measured. The X-ray attenuation analysis indicated that the thin film with 2 µm thickness attenuated 10% of the photon flux with 20 to 29 KeV of energy, while the sample with 21 µm thickness attenuated 60% of the photon flux.

## 1. Introduction

The development of new ionizing radiation technologies for industrial, medical and defense applications is an important research topic with major contributions to the improvement of quality of life. However, radiation protection is required for the safety of operators, patients and other electronic systems located near ionizing radiation sources. For the manufacturing of electromagnetic shields, high-Z metals like lead, tungsten and barium are chosen [1]. The intensity attenuation of ionizing radiation when it penetrates a metal is described using the Beer–Lambert law. The process of attenuating ionizing radiation is based on the photon absorption mechanism of the metal electrons via photoelectric [2] and Compton scattering effects [2,3]. Metals are good attenuators of ionizing radiation, but they have some disadvantages, such as the following: they are heavy, some can be toxic and metal plates are rigid. Therefore, metals are sometimes difficult to use as electromagnetic shields in various industrial and medical fields. To overcome these difficulties, numerous studies have been recently performed on the interaction of ionizing radiation with metallic powders. These studies showed that in the interaction between ionizing radiation and metallic powders, the photoelectric effect probability occurrence is higher because the photons of the ionized radiation are reflected and absorbed multiple times between the metallic particles, resulting in further dissipation of the photon energy [3]. Therefore, metallic nanoparticles are more absorbent of the ionized radiation than metallic microparticles [4]. Experimental studies have been carried out on the integration of metal powders into polymer materials [5] and textiles [6]. The results of this research have shown that metallic powders in combination with these materials allow for the development of superior shields against ionizing radiation with better physical properties than metallic plates. Knowing that carbon-fiber-reinforced polymer composites (CFRPs) are lightweight and inexpensive materials, with excellent mechanical and thermal properties and chemical resistance [7], which could be an alternative to metals for numerous structural uses [8], the possibility of using them as electromagnetic shields was evaluated. CFRPs have strength and stiffness properties that, in many cases, exceed those of metals. 

Also, CFRPs are transparent in the ionizing radiation domain [9]. To use CFRPs as electromagnetic shields, they must be made opaque for ionizing radiation via the deposition of a layer of metallic nanopowder. The generation and deposition of metallic nanopowders using laser ablation and plasma methods are performed at high temperatures [10,11], and the composite materials are degraded at temperatures above 200 °C [12]; therefore, this research assessed the possibility of simultaneously synthesizing and depositing tungsten oxide nanopowders onto the surface of CFRPs using a new microwave plasma generation method in atmospheric air. This new method uses the direct interaction of microwaves with an electrically insulated tungsten wire to create hot plasma and metallic nanopowders. So far, many experimental microwave devices have been developed to be used for liquid–gas heated decomposition, plasma generation or the sintering of metal powders. In 2000, Whittaker et al. [13] conducted experimental research on the interaction between microwaves and metallic powders mixed into a liquid medium. Using a commercial microwave oven, they observed that during interaction with microwaves, the metallic particles generated electrical arcing, resulting in the heating of the liquid.

In 2001, Chen et al. [14] performed studies on microwaves’ interaction with metals, ceramics and metal–ceramic composites. Using an experimental single-mode device, small samples from metals, ceramics and metal–ceramic composites were heated under a nitrogen atmosphere. During the exposure of the samples to microwaves, their temperature did not exceed the value of 1000 °C.

In 2009, Mondal et al. [15] observed that during the interaction of the microwaves with metallic powders with different dimensions, the metallic particles were heated. 

Using a multimode microwave furnace, the authors exposed copper powders with dimensions between 6 µm and 385 µm to a microwave field. Following this experiment, it was highlighted that metallic particles with 6 µm dimensions reached a temperature value of 1200 °C, while the particles with 385 µm dimensions reached a value of 800 °C.

In 2018, Yukun Feng et al. [16] introduced into a microwave oven a quartz glass cylinder, where they placed metallic paper clips and gaseous acetone. Following the interaction between the microwaves and metallic paper clips, electric sparks were generated, and the acetone was decomposed. Other similar experiments were performed by Popescu S. et al. [17]. Using a microwave device, two titanium pieces brought into contact during irradiation with microwaves generated plasma and metallic particles in atmospheric air. During the interaction between the microwaves and metallic pieces, the electronic temperature of the plasma reached the value of 0.4 eV.

Compared to other experiments or microwave devices, the new method described in this paper offers a high vaporization rate of the metallic wire without the metallic wire being in contact with other electrodes. In this way, the contamination of the metal vapors that can appear from other contact metals used as electrodes is avoided, and during the interaction of the microwaves with the metallic wire, much higher electronic temperatures of the plasmas are reached. In order to characterize the plasma and metallic powders, a series of investigations were carried out.

Plasma diagnostics was performed using the optical emission spectroscopy method. After the deposition process, the structure, shape and dimensions of the tungsten oxide particles deposited on the CFRP samples were analyzed by grazing incidence X-ray diffraction (GIXRD), scanning electron microscopy (SEM) and transmission electron microscopy (TEM). It was also found that the surface of the CFRP samples was not degraded during the microwave plasma deposition process. 

## 2. Materials and Methods

**Figure 1:**
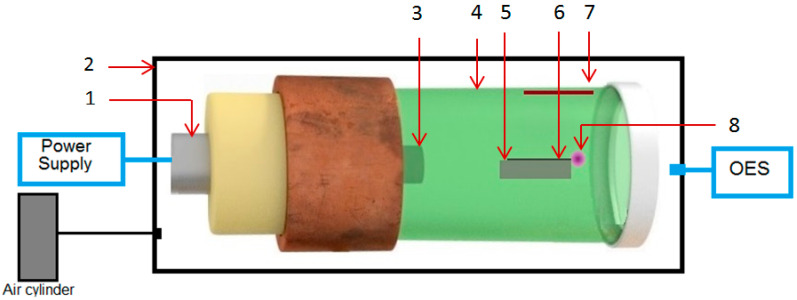
Design of the experimental setup: 1—commercial magnetron; 2—pressure chamber; 3—magnetron antenna; 4—waveguide cavity; 5—ceramic support; 6—metallic wire; 7—deposition substrate; 8—plasma.

Microwaves are non-ionizing radiation having a frequency between 300 MHz and 300 GHz, located between the radio and IR regions of the electromagnetic spectrum. In literature, two main physical processes are described as taking place when microwaves interact with matter: absorption and reflection [18]. Dielectric materials are heated as the result of the absorption of microwaves, while metals reflect microwaves. Using a commercial magnetron (800 W microwave power, frequency = 2.45 GHz) coupled through an antenna to an electromagnetic waveguide, a new device used to generate plasmas from metallic wires in atmospheric air (Figure 1) was designed. The waveguide was constructed to match the TM_011_ (transverse magnetic) propagation mode, its role being to focus the electric field of the microwaves into a single point located on the cylindrical cavity axis. If the magnetron generates a microwave power of 800 W, in the focal point of the waveguide there will be 8 MW/cm^2^ [19]. When an electrically insulated metallic wire is placed with one end in the focal point of the waveguide and irradiated, it will emit electrons through the field emission effect [20]. In this case, the metallic wire is the negative electrode while the waveguide cavity is the positive electrode. The electric voltage induced by microwaves in a tungsten wire with a 0.5 mm diameter and a length of 0.5 cm will reach up to 71 kV [21]. If a metallic wire placed in a gas atmosphere is exposed to such a high power density of microwaves, then a plasma will be generated.

**Figure 2:**
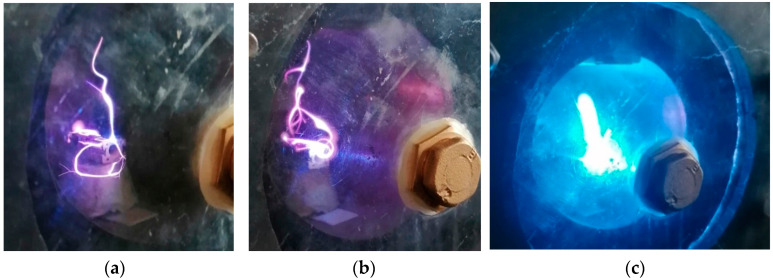
(**a**–**c**) Photographs of the generation and evolution of the microwave discharge at an interval of one second.

**Figure 3:**
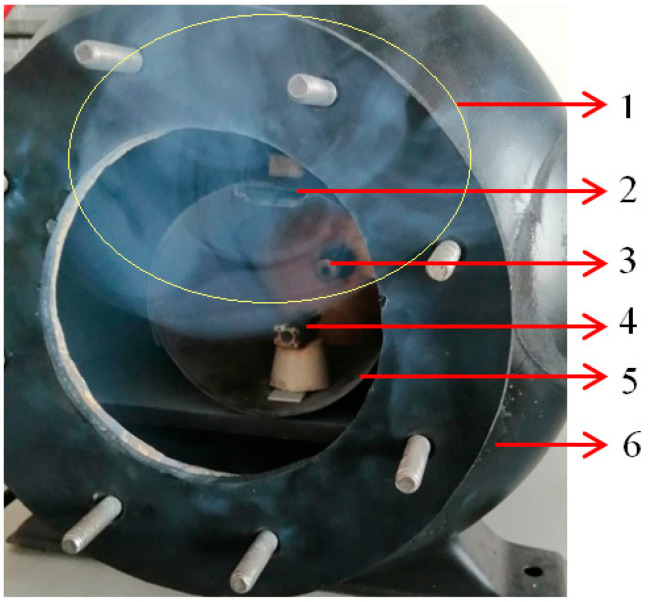
Generation of tungsten oxide nanoparticles during microwave discharge process: 1—tungsten oxide cloud; 2—metallic support; 3—magnetron antenna; 4—metallic wire; 5—TM_011_ waveguide; 6—pressure chamber.

Figure 2a displays an image of the plasma initiation process of the microwave discharge immediately after the magnetron power is turned on. If the metallic wire interacts with microwaves in a gas atmosphere, the atoms of gas are ionized, and the metallic wire is strongly heated by collisions with ions. In Figure 2b, one can observe that in the focal point of the waveguide, the plasma begins to increase in volume following the initiation of the thermionic effect; in this stage, metal ions begin to appear. In Figure 2c, one can observe that the plasma was concentrated in the focal point of the waveguide where the metallic wire was strongly heated. In this stage, the thermionic process is predominant, and the metallic wire is vaporized. The metallic vapors were mixed with the gas heated by the plasma and expanded into the entire volume of the waveguide, then condensing on the walls of the waveguide (Figure 3).

**Figure 4:**
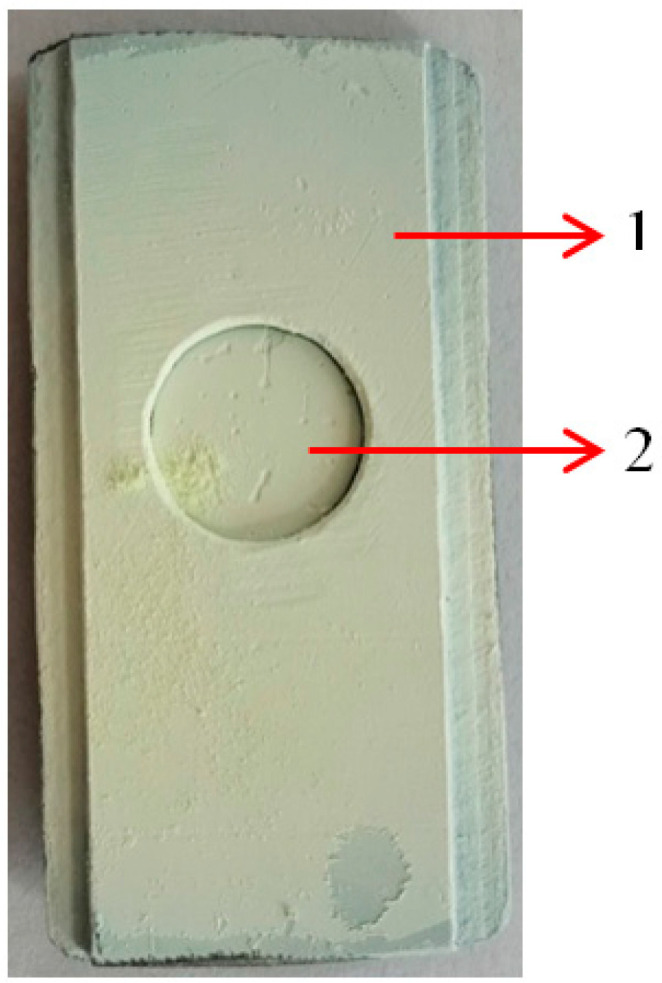
The tungsten oxide layer with 21 μm thickness deposited on the surface of the CFRP sample: 1—metallic support; 2—CFRP sample after deposition process.

If the microwave discharge is performed in a gas containing oxygen such as CO_2_ [22] or air [23], metal oxide nanoparticles are formed. If a CFRP substrate or other material is placed inside the waveguide above the zone where the plasma is generated (Figure 3), the metal oxide vapors will also be deposited on that substrate (Figure 4).

Before starting the process of depositing the metal oxide, two CFRP samples and a tungsten wire were prepared. The samples had the dimensions of 1.5 cm × 1.5 cm and a thickness of 2 mm. The tungsten wire was cut at 5 cm length and was 0.5 mm in diameter. The experiment started with the tungsten wire being placed along the cylindrical cavity symmetry axis with one tip located in the focal point of the waveguide. A CFRP sample was placed at a 5 cm distance above the focal point of the cavity (see Figure 1). 

The microwave power source was turned on, and then the power of the microwaves was increased until the metallic wire was ignited. The first sample was exposed to the microwave discharge for 10 s, and the second sample for 50 s. 

After the deposition of the tungsten oxide thin film on the CFRP sample, the microwave power source was turned off. During the microwave discharge, the plasma was investigated using the optical emission spectroscopy (OES) method [24,25] with an Ocean Optics USB 2000++ spectrometer (Ocean Optics Inc., Orlando, FL, USA). 

The optical emission spectrum of the plasma generated by the metallic wire interacting with microwaves was recorded with a 10 ms integration time. Before the plasma was analyzed, the spectrometer was calibrated using the cValSpec system, which utilizes the emission of four distinct line lamps covering the spectral range from 200 to 900 nm. To convert the spectrometer signal from arbitrary units to SI radiance units, a radiometric calibration was performed using the laboratory spectral radiance standard OL455 from Optronic Lab. Orlando, FL, USA. This standard employs a uniform radiance source with adjustable luminance levels, ensuring accurate calibration across the measured spectral range. To identify each chemical element from plasma and correct the emission spectrum of the plasma, the experimental results were compared to three of the most intense spectral lines for each element from the National Institute of Standards and Technology (NIST) database [26]. The optical resolution according to the datasheet of the Ocean Optics USB 2000 + spectrometer is FWHM ~ 0.1 nm [27].

To determine the chemical composition of the tungsten oxide generated in microwave discharge, the samples deposited with metallic nanopowders were investigated by X-ray photoelectron spectroscopy (XPS) with an ESCALAB 250Xi instrument (Thermo Fisher Scientific, Warrington, UK). This instrument uses an electrostatic analyzer, with an X-ray source and Al Kα radiation anode (hν = 1486.6 eV). The spectra were acquired with a pass energy of 20 eV and a step size of 0.1 0 eV (higher resolution) for narrow scans and a pass energy of 100 eV and a step size of 0.5 eV (lower resolution) for extended scans.

**Figure 5:**
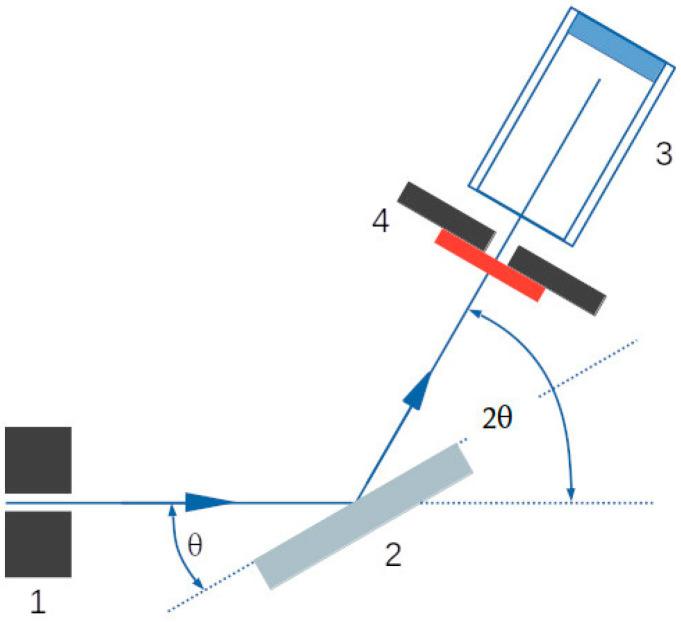
Sketch of X-ray diffraction and θ-2θ coupling. 1—collimator; 2—NaCl crystal; 3—Geiger Muller detector; 4—CFRP sample.

To determine the X-ray attenuation induced by the tungsten oxide nanopowder, the CFRP samples deposited with tungsten oxide were exposed to energy intervals between 21.03 KeV and 29.32 KeV using a Leybold X-ray apparatus (Figure 5), which uses an X-ray tube to produce X-rays. The X-ray photons were collimated and then diffracted by a NaCl crystal placed on a rotating platform. The crystal and the counter were rotating in a θ-2θ coupling. 

Bragg’s law (Equation (1)) was used to describe quantitatively the dependence of the X-ray wavelength diffracted by the NaCl crystal on the incident angle θ.

λ = 2 d sin (θ) (1)

where λ—the wavelength of the X-ray photon; d—the inter-planar distance of the NaCl crystal (d = 2.82 Å); θ—the angle made by the incident beam with the crystal’s atomic planes. 

According to Bragg’s law, for different angles of incidence detection, photons with different energies will be diffracted by the probe and directed into the detector. Only the photons with a specific energy would constructively interfere so that the detector positioned at a specific angle would record the signal, in accordance with the formula stated above. In other words, the increase in the θ angle will correspond to a decrease in the detected photon’s energy.

## 3. Results

**Figure 6:**
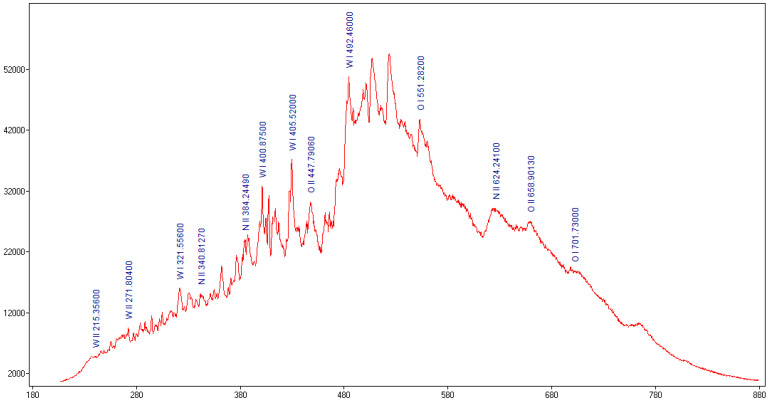
The optical emission spectrum of plasma generated by the tungsten wire when exposed to 800 W microwave power in atmospheric air.

Using Span V.1.7 Spectrum Analyzer software [28], the recorded optical emission spectrum of the microwave discharge was analyzed, and the result is displayed in Figure 6. One can observe that during the interaction between microwaves and the tungsten wire, a plasma is generated, which emitted spectral lines in UV-VIS-NIR domain. These spectral lines correspond, in agreement with the spectral lines from the NIST database, to metallic excited atoms and ions, namely WI and WII, and gas excited atoms and ions, namely OI, OII, NI and NII [29].

**Figure 7:**
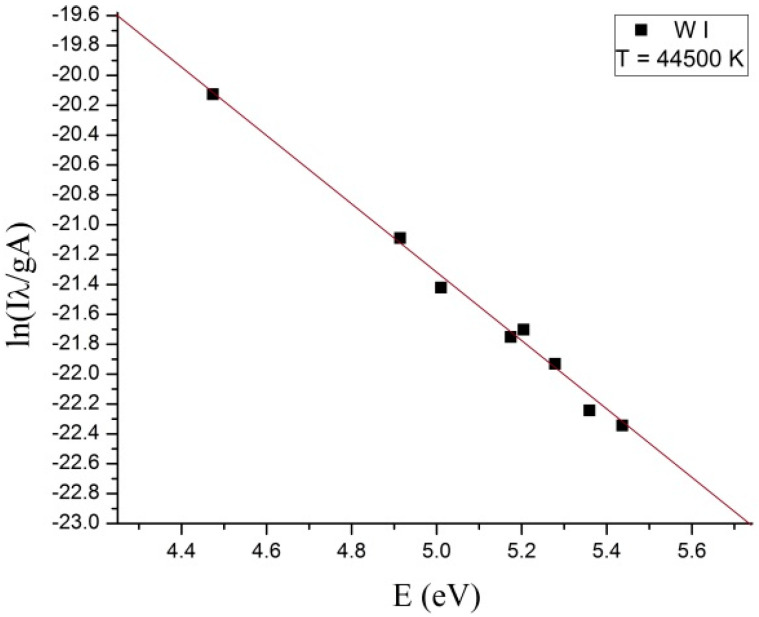
Boltzmann plot for WI in air at normal pressure.

To estimate the electronic temperature of the plasma for WI and WII ionic species, the Boltzmann plot method was used, which assumes that local thermodynamic equilibrium (LTE) is met within the plasma. The electronic Boltzmann plot from which the excitation temperature of WI species was estimated is shown in Figure 7. From Figure 7, we noticed that the obtained plasma is thermal [30] for the excited neutral atom species from microwave discharge. 

**Figure 8:**
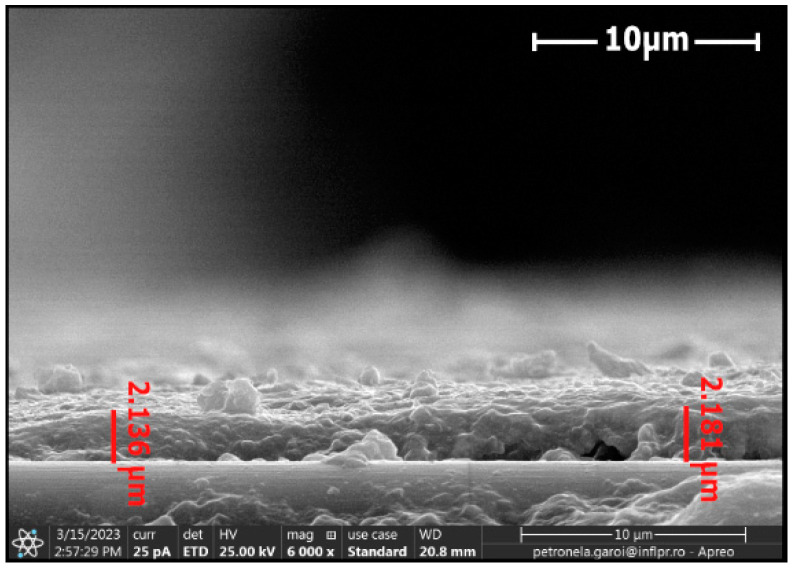
Cross section of the tungsten oxide thin film deposited for 10 s on the CFRP sample.

**Figure 9:**
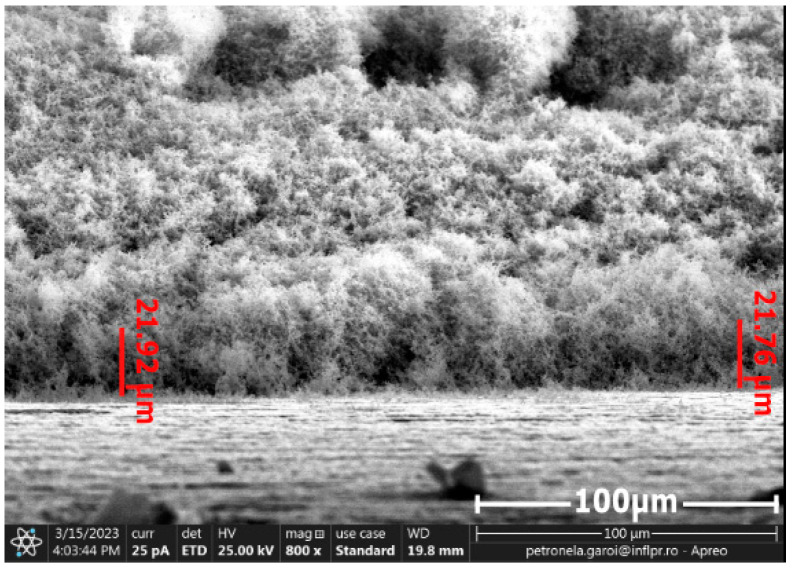
Cross section of the tungsten oxide thin film deposited for 50 s on the CFRP sample.

The energetic domain presented on the X-axis of the Boltzmann plot corresponded with the frequency of the spectral lines selected for determining the electronic excitation temperature of the excited neutral atom species, where for ln(IλgA), I—intensity of the spectral lines; λ—wavelength of the spectral lines; A—transition probability; g—the statistical weight of the upper energy level; excitation energy (eV)—energy level of upper state. To determine the dimension of the tungsten oxide particles and thicknesses of the layers of the tungsten oxide deposited on the surface of the CFRP samples, we used scanning electron microscopy (SEM) investigations. Figure 8 and Figure 9 display the cross sections of the tungsten oxide thin films deposited on the CFRP samples. Figure 8 was scanned at 6000× magnification, and Figure 9 was scanned at 800× magnification. 

**Figure 10:**
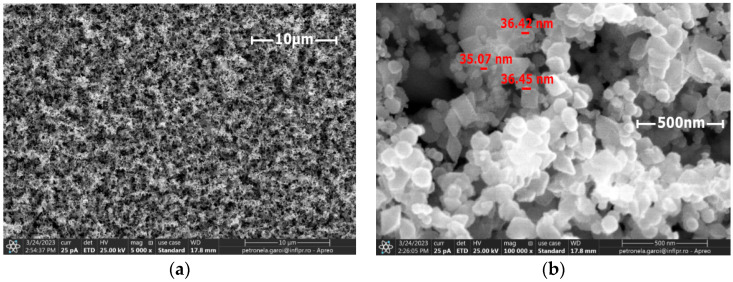
SEM images of the sample deposited with a 2 µm thick tungsten oxide nanopowder; (**a**) general appearance; (**b**) high-resolution image of the nanoparticles.

**Figure 11:**
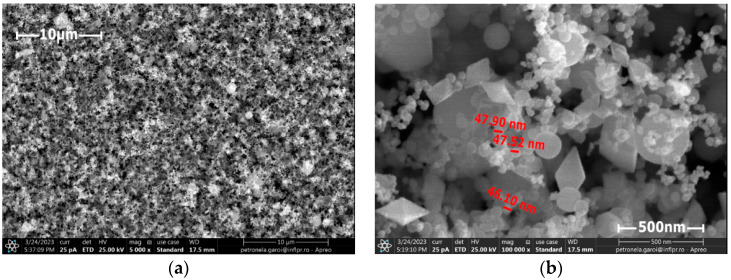
SEM images of the sample deposited with a 21 µm thick tungsten oxide nanopowder; (**a**) general appearance; (**b**) high-resolution image of the nanoparticles.

For the first CFRP sample exposed for 10 s to microwave discharge, a tungsten oxide thin film of 2 µm thickness was deposited (Figure 8), and for the second CFRP sample (Figure 9) exposed to 50 s of microwave discharge, a tungsten oxide thin film of 21 µm thickness was deposited. Figure 10 and Figure 11 display the SEM images for the two samples. Figure 10a and Figure 11a were scanned at 5000×, and Figure 10b and Figure 11b were scanned at 100,000×.

The SEM analysis of the deposited layers showed that the nanoparticles were uniformly distributed on the CFRP samples and that they have various sizes and shapes, such as rounded, polyhedral and rhombohedral ones. 

TEM analysis provided us with a better understanding of the morphology and crystallinity of the synthesized nanostructures, as well as the local phase composition. 

**Figure 12:**
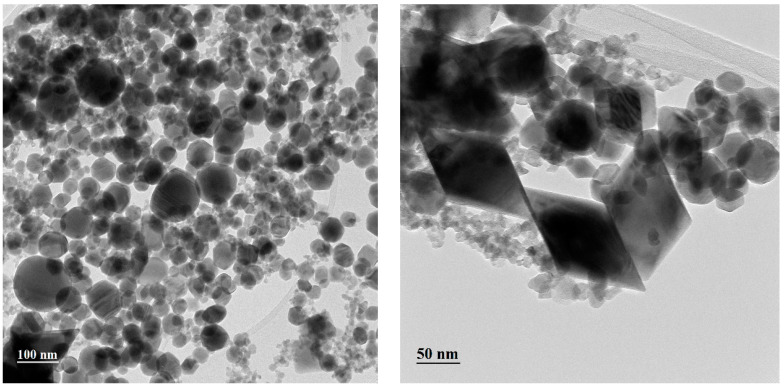
BFTEM image of the deposited sample.

**Figure 13:**
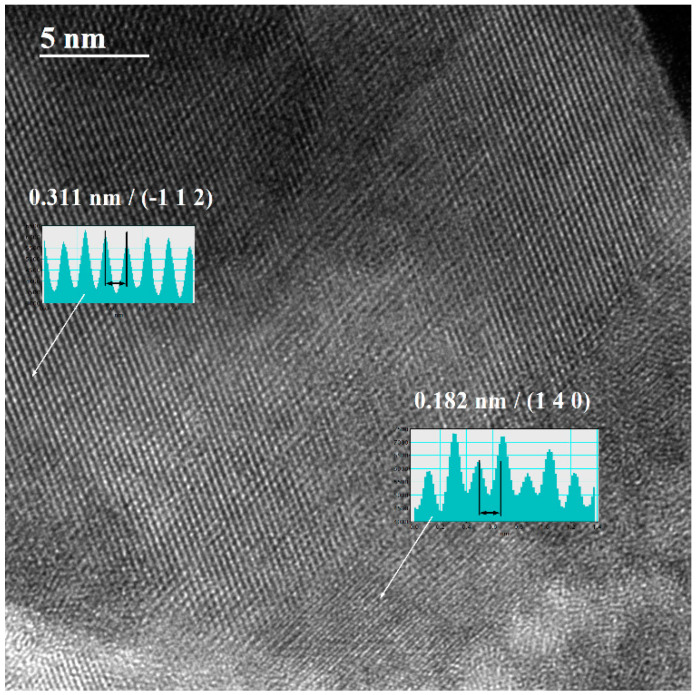
HRTEM image of the deposited sample; the inserts indicate the inter-planar spaces.

The bright-field TEM (BFTEM) images acquired from the thickest WO_3_ film deposited are presented in Figure 12. Various shapes of nanoparticles are identified, from spherical (majority) to polyhedral and rhombohedral, with a bimodal distribution, starting from 5 nm to up to 100 nm. The nanoparticles of WO_3_ are single crystals and exhibit various orientations, as one can observe in Figure 13, where a high-resolution TEM image is presented.

**Figure 14:**
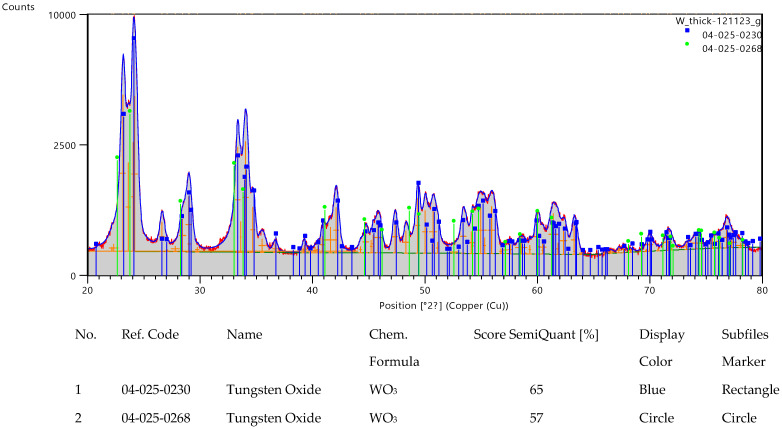
Analysis of the GIXRD patterns acquired from the tungsten oxide powders deposited for 50 s.

**Figure 15:**
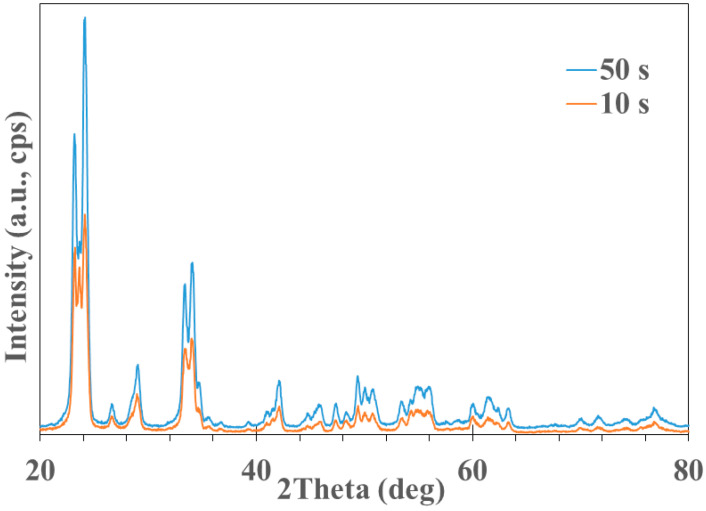
GIXRD patterns acquired from deposited nanostructures (red line—first sample; blue line—second sample).

Figure 14 displays the structural analysis of the tungsten oxide diffraction patterns performed with HighScore Plus 4.1 software from Panalytical (Almelo, the Netherlands). Very good matches for the acquired patterns both in terms of peak positions and relative intensities were found for WO_3_ monoclinic (reference code 04-025-0230, matching score of 65 and semi-quantitative fraction of 68%, blue markers) and WO_3_ tetragonal (reference code 04-025-0268, matching score of 57 and semi-quantitative fraction of 32%, green markers). The XRD patterns acquired from the two WO_3_ samples are displayed in Figure 15. One can note that the patterns corresponding to the thicker film are more intense. However, the peak positions and relative intensities are very similar, indicative of the same crystalline structure.

**Figure 16:**
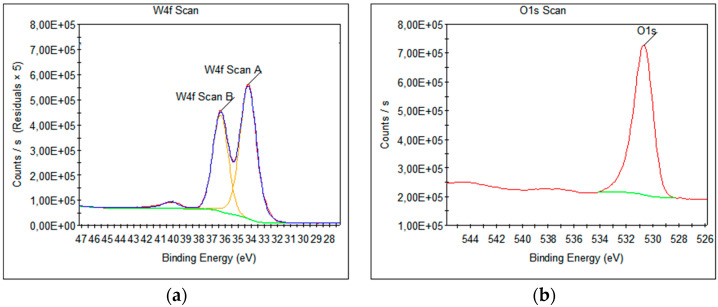
High-resolution XPS spectra acquired from WO_3_: (**a**) W 4f and (**b**) O 1s; green line is a Shirley-type background, yellow lines are the deconvolution of the W 4f peak, the blue line is the sum of the fitted peaks and the red line is the experimental spectrum.

The analyzed survey XPS scans and high-resolution spectra showed the presence of W4f (Figure 16a) and O1s (Figure 16b) from WO_3_. The same chemical states of W4f, namely W4f7/2 (scan A) and W4f5/2 (scan B), and O1s were identified by high-resolution XPS (HR-XPS) scans.

XPS analyses showed the quality of WO_3_ and the chemical composition. Table 1 presents the results of the XPS investigations, including the peak binding energy and the full width at half maximum (FWHM), which are useful indicators of chemical state changes and physical influences, and the atomic percentage. 

**Figure 17:**
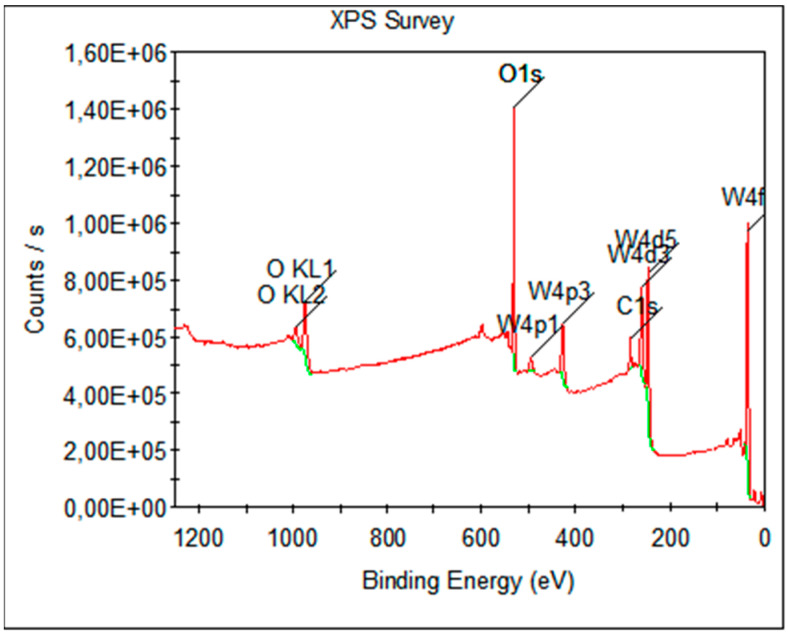
XPS survey spectrum of WO_3_ powder; green lines are Shirley-type backgrounds in the peaks’ regions.

The general spectrum (survey XPS) of WO_3_ is shown in Figure 17, where peaks corresponding to C 1s, O 1s, W 4p, W4d and W4f were identified for the investigated sample. The presence of photoelectron signals from WO_3_ and carbon contamination at the binding energy value of 284.8 eV were observed. It must be said that no other metallic elements were identified. 

**Figure 18:**
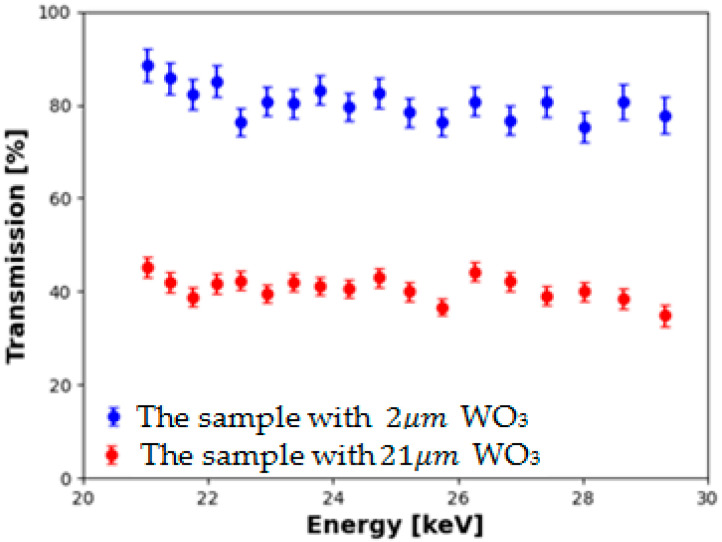
X-ray transmission vs. photons energy. Red and blue symbols correspond to samples having 2 µm and 21 µm thick tungsten oxide layers, respectively.

In Figure 18, the transmission intensity is represented as a function of the X-ray photon’s energy. During the X-ray investigation, the transmission of the CFRP sample was taken as a reference (100%) in the X-ray scanning process. The figure shows a clear attenuation of the X-ray photons with a value of 20.33 ± 4.86% for the sample deposited with 2 μm thickness WO_3_ and 59.37 ± 2.49% for the sample deposited with 21 μm thickness WO_3_.

## 4. Discussion

Tungsten is an abundant metal from nature with unique physical properties. Currently, tungsten oxides are used in various technological branches. The oxidation of tungsten depends on the temperature and the oxygen concentration [31]. In general, tungsten is mostly a non-reactive element. To obtain a tungsten oxide layer with a thickness of ~3 mm, a tungsten piece must be exposed for ~15 h in air at 1100 °C [32]. Therefore, it is currently difficult to manufacture tungsten oxides at an industrial scale. Using a microwave generator (Figure 1) we created a plasma and WO_3_ oxide from a tungsten wire in air at atmospheric pressure. During the microwave discharge, metallic ions (WI, WII) and gas ions (OI, OII, NI, NII) were generated, and the metallic wire tip located in the waveguide focal point was completely vaporized.

From the Boltzmann plot, it was observed that a thermal plasma was obtained for WI atoms, and the electronic temperature of the plasma reached a high value of ~4 eV. At this electronic temperature value of the plasma, the WO_3_ oxide was practically formed instantly. Considering that in this microwave discharge, all of the volume of the tip of the tungsten wire is vaporized, we can infer that the tungsten wire reached a minimum of 5555 °C temperature, which corresponds to the boiling point of tungsten [33]. To identify the zone where the WO_3_ oxide was formed, plasma was initiated for a short time, and then EDS analysis was performed on the tip of the tungsten wire.

**Figure 19:**
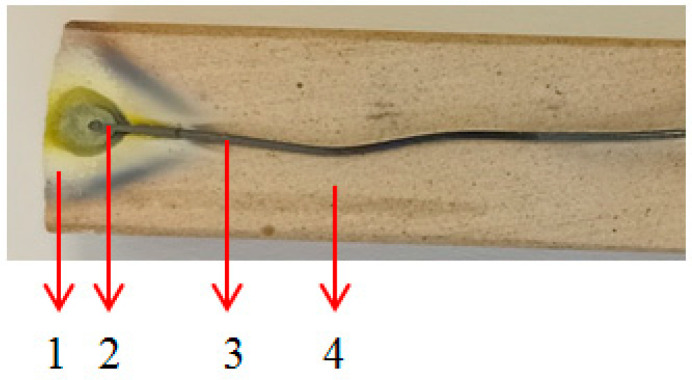
Image of the tungsten wire after plasma initiation process: 1—WO_3_ oxide; 2—tip of the wire; 3—unaffected tungsten wire; 4—ceramic support.

Figure 19 displays an image of the tungsten wire after the plasma initiation process.

Figure 19 shows that the tungsten wire is vaporized only in the focal point of the waveguide and the metallic wire is not heated in volume. To perform EDS analysis, an Apreo S microscope from Thermo Fisher Scientific (Eindhoven, the Netherlands) with an energy-dispersive X-ray spectroscopy (EDS) system, fixed silicon detector and integrated Peltier element as a cooling system was used. For EDS, the used beam spot was 6.5–7 µm in diameter, the working distance was 10 cm and the dead time during signal collection was 30 s.

It was also operated at 10 kV acceleration voltage and 6.3 pA electrical current. EDS analysis performed in the tip region of the wire found that traces of oxygen were present. The results of EDS investigations, mass percentage and atomic percentage, are presented in Table 2.

Therefore, after the plasma initiation process, the tungsten vapors react with oxygen from the air and form WO_3_ [34], and then the WO_3_ nanoparticles are collected on the waveguide wall. The rate of vaporization of the tungsten wire was ~70 mg/s at 800 W microwave power. The dimension of the WO_3_ particles deposited on the CFRP samples was between 5 nm and 100 nm. The mechanism of nanoparticle formation is very complex and includes several physical processes [35]. These depend on several parameters such as the plasma temperature, the kinetic energy of the ions, and the gas in which the discharge is ignited [36]. Given that the microwave discharge is ignited in atmospheric air, the hot metal ions will interact with oxygen atoms, forming WO3 molecules that will nucleate the growth of various nanocrystalline structures.

Even if the first sample was deposited with a 2 μm tungsten oxide layer in 10 s plasma exposure time and the second sample was deposited with 21 μm tungsten oxide in 50 s exposure time, the vaporization process of the tungsten wire is uniform in time. To generate metallic oxide powders using this method, it is necessary to wait a few seconds from the ignition of the wire until it reaches a constant generation rate of the oxide nanoparticles. Then, the WO_3_ nanoparticles will be deposited on the substrate sample layer upon layer. Given that the value of the plasma temperature is very high, after 50 s from the initiation of the microwave discharge, the CFRP substrate sample reached ~70 °C.

**Figure 20:**
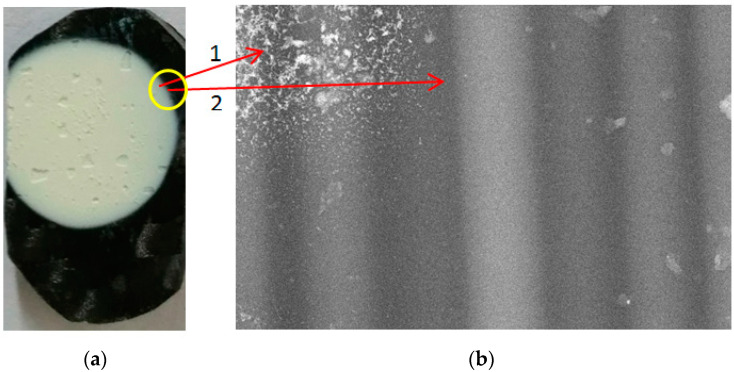
The images of the second sample: (**a**) general image of the CFRP sample; (**b**) SEM image of the CFRP sample scanned at the interface between the deposition area and the clean area.

Figure 20a displays an image of the CFRP sample deposited for 50 s, and Figure 20b displays an SEM image of the same sample acquired at the interface between the deposited area and the area under the metal support (Figure 4) where the tungsten oxide particles did not penetrate (inside of the yellow circle zone).

Figure 20b was scanned at 5000× magnification. Point 1 corresponds to an area with deposited tungsten particles, while point 2 corresponds to a clean area. From these images, it is observed that the structure of the CFRP sample is not affected during the deposition process. The temperature of the CFRP sample remained low because this was cooled by the air mixed with metallic particles located between the plasma and the walls of the waveguide.

The thickness of the deposited nanoparticles layer depends on the used deposition time. However, the maximum thickness reached in our present experimental setup is limited by the length of the W wire located in the cavity nodal point, where the electrical field is high enough to ignite a plasma. Once the W wire from the nodal point is consumed, the plasma is stopped, and a maximum layer thickness value is reached. If a mechanical system that can continuously advance the W wire at a rate equal to the evaporation rate is designed and implemented, then the thickness of the deposited nanopowders could keep linearly increasing with the deposition time for as long as one wishes.

Regarding the attenuation of X-rays by tungsten oxide nanoparticles, it was observed that the sample deposited with 2 µm tungsten oxide nanopowder thickness attenuated 10% of the X-ray photon flux, while the sample deposited with a 21 µm thick tungsten oxide nanopowder thickness attenuated 60% of the X-ray photon flux.

Therefore, the attenuation of the X-rays by the tungsten oxide nanopowder depends on its thickness. Using this microwave deposition technique, one can improve the attenuation of the ionizing radiation of the CFRPs following two directions: through the mixing of the tungsten oxide nanopowder with an epoxy solution (base resin from composite materials) or through the coverage of the surface of CFRPs with base resin and then the deposition of the metallic oxides directly on the CFRPs. Using the base resin before the deposition process offers a strong adhesion between metallic oxides and the CFRP substrate. At an industrial scale, this device can generate metallic oxide powders in a continuous regime with the addition of a roll with metallic wire and a mechanical part that continuously advances the metallic wire in the focal point of the waveguide.

This deposition method can be used in the aerospace domain and medical industry to deposit oxide metal powders on the surface of parts made of materials that have low melting points and complex shapes. In this way, an improvement in performances related to radiation and electromagnetic compatibility requirements can be obtained.

## 5. Conclusions

Using this simple and inexpensive microwave deposition technique, tungsten oxide nanocrystalline powders were obtained in atmospheric air. The SEM analysis of the tungsten particles showed that during microwave discharge, tungsten oxide crystals with dimensions between 5 nm and 100 nm were generated. TEM analysis highlighted that high-purity stoichiometric oxide single crystals without defects were generated during microwave discharge.

Even if the plasma temperature reaches very high values, the deposition of the metallic oxide can be performed directly on the surface of a CFRP or other materials with low melting points without affecting the materials.

From an X-ray attenuation investigation, we observed that the sample deposited with a 2 µm tungsten oxide nanopowder thickness attenuated 10% of the X-ray photon flux with energies between 20 keV and 29 keV, while the sample deposited with a 21 µm thick tungsten oxide nanopowder attenuated 60% of the photon flux in the same energy domain. Using CFRP as a substrate in the deposition process with tungsten oxide nanopowder, a new composite material that exhibited high X-ray attenuation was obtained.

## Figures and Tables

**Table 1:** XPS results for WO_3_.

Name	Peak BE (eV)	FWHM (eV)	Atomic (%)
W4f	35.98	5.33	85.75
O1s	530.75	2.71	14.25

**Table 2:** EDS analysis of the tip of the tungsten wire.

Surface Element Line	Weight %	Atomic %
W L	98.53	85.35
O K	1.47	14.65

## Data Availability

Data are contained within the article.
